# Strigolactone Regulates Anthocyanin Accumulation, Acid Phosphatases Production and Plant Growth under Low Phosphate Condition in *Arabidopsis*

**DOI:** 10.1371/journal.pone.0119724

**Published:** 2015-03-20

**Authors:** Shinsaku Ito, Tomoko Nozoye, Eriko Sasaki, Misaki Imai, Yuh Shiwa, Mari Shibata-Hatta, Taichiro Ishige, Kosuke Fukui, Ken Ito, Hiromi Nakanishi, Naoko K. Nishizawa, Shunsuke Yajima, Tadao Asami

**Affiliations:** 1 Graduate School of Agricultural and Life Sciences, The University of Tokyo, 1-1-1 Yayoi, Bunkyo, Tokyo, Japan; 2 Department of Bioscience, Faculty of Applied Bioscience, Tokyo University of Agriculture, 1-1-1 Sakuragaoka, Setagaya, Tokyo, Japan; 3 Gregor Mendel Institute of Molecular Plant Biology, Vienna, Austria; 4 Genome Research Center, Tokyo University of Agriculture, 1-1-1 Sakuragaoka, Setagaya, Tokyo, Japan; 5 Department of Biochemistry, King Abdulaziz University, Jeddah, Saudi Arabia; University of Delhi South Campus, INDIA

## Abstract

Phosphate is an essential macronutrient in plant growth and development; however, the concentration of inorganic phosphate (Pi) in soil is often suboptimal for crop performance. Accordingly, plants have developed physiological strategies to adapt to low Pi availability. Here, we report that typical Pi starvation responses in *Arabidopsis* are partially dependent on the strigolactone (SL) signaling pathway. SL treatment induced root hair elongation, anthocyanin accumulation, activation of acid phosphatase, and reduced plant weight, which are characteristic responses to phosphate starvation. Furthermore, the expression profile of SL-response genes correlated with the expression of genes induced by Pi starvation. These results suggest a potential overlap between SL signaling and Pi starvation signaling pathways in plants.

## Introduction

Phosphate (Pi) is an essential macronutrient for plants and plays an important role in major metabolic processes; however, a large component of Pi in soil is present in organic compounds, meaning that much of the Pi is insoluble and not readily available to plants. Thus, plants have developed various alternative systems to adapt to low Pi, including symbiotic interactions with mycorrhizae, the secretion of acid phosphatases, root hair elongation, the accumulation of anthocyanins, reduced plant growth, and the up-regulation of phosphate starvation-inducing (PSI) genes [1].

PSI genes are commonly used as markers for the Pi starvation response. Purple acid phosphatases (*PAP*s), which are important members of PSI gene families, hydrolyze organic phosphates into Pi. Intracellular acid phosphatases play an important role in P homeostasis *in planta*, whereas secreted acid phosphatases are intimately related to the utilization of organic phosphate in soils, which cannot be readily assimilated by plants in their organic form [2]. *PHOSPHATE2* (*PHO2*), which encodes a ubiquitin-conjugating E2 enzyme (UBC24), functions as a repressor and prevents excessive accumulation of Pi by controlling Pi uptake and root-to-shoot Pi translocation[3,4]. The *PHOSPHATE TRANSPORTER1* (*PHT1*) family, a group of nine closely related members, mediates external Pi uptake. Transcriptional analysis of *PHT* genes indicated that *PHT1* is highly expressed under low-Pi conditions [5,6]. The non-protein encoding gene *IPS1* (*INDUCED BY PHOSPHATE STARVATION1*) is also considered a PSI gene and contains a motif with sequence complementarity to miR-399, a Pi starvation induced miRNA that regulates *PHO2* transcription [7].

Strigolactones (SLs) are terpenoid lactones produced by many plant species and have various roles including: promoting the germination of parasitic weeds, signaling hyphal branching in mycorrhizal fungi, and functioning in shoot branching, root morphology, and secondary growth [8–12]. To date, several mutants with aberrant branching patterns such as *more axillary growth* (*max*) in Arabidopsis, *semi-dwarf* (d) in rice, *decreased apical dominance* (*dad*) in petunia and *ramosus* (*rms*) in pea, have been characterized as SL biosynthesis and/or signaling mutants. At present, two carotenoid cleavage dioxygenases (AtMAX3 and AtMAX4), a carotenoid isomerase (AtD27), and a cytochrome P450 (AtMAX1: CYP711A1) are known to be involved in the biosynthesis of SLs in *Arabidopsis*. *AtMAX2* encodes an F-box protein and a petunia (PhMAX2) and rice (D3) homologs of AtMAX2 interact with D14, a putative SL receptor, in the presence of SL [13,14,15]). Recently, D53, which encodes a substrate of the SCF^D3^ complex, was reported as a repressor of SL signaling in rice [16,17] and it was proposed that the regulation of SL signaling was triggered by the proteasome-mediated degradation of D53 by the SCF^D3^ complex. In the SL biosynthetic pathway, D27 catalyzes the isomerization of all-*trans*-ß-carotene to 9-cis-ß-carotene, which is sequentially cleaved by CCD7 to form 9-*cis*-ß-apo-10’-carotenal and then by CCD8 to yield the carlactone (CL) [18]. A rice homolog of AtMAX1 acts as a CL oxidase to stereoselectively convert CL into *ent*-2’-*epi*-5-deoxystrigol, which is the major SL in rice [19]. *max1-1* and *max2-1* mutants show the aberrant branching pattern, defect of normal root formation, delay of leaf senescence and so on [12]. These phenotypes of *max1-1* mutant were rescued by SL treatment, while those of *max2-1* mutant were not. That is, *max1-1* is SL responsive mutant and *max2-1* is SL insensitive mutant. In addition, only *max2*, but not other SL mutants, shows the phenotypes of reduced germination efficiency, longer hypocotyls, and hooked epinastic cotyledons [14,20,21].

SLs have been shown to regulate diverse physiological phenomena including shoot branching, root hair elongation, lateral root formation, and PSI gene expression [22–25]. Pi deficiency also regulates these phenomena and increases SL levels. Furthermore, SLs are involved in root hair development under low-Pi conditions, because the number of root hairs was reduced in SL biosynthesis (*max4-1*) and signaling (*max2-1*) mutants under low Pi condition during the early stage of seedling development [25]. These observations suggest that SLs could be a mediator of low phosphate responses; however, the effects of SLs on typical phosphate starvation responses are poorly understood.

In this study, we evaluated the effects of SLs on phenomena induced by phosphate starvation under low- and high-Pi conditions; we discovered that SLs induce various phosphate starvation-inducing events, induction of anthocyanin accumulation and acid phosphatase production, and the reduction of plant growth, in wild-type (WT) and SL-biosynthetic mutants but not in SL-insensitive mutants. Furthermore, SL-biosynthetic and-insensitive mutants showed altered responses to Pi starvation in comparison with WT plants. RNA sequencing (RNAseq) of the SL biosynthetic-mutant (*max1-1*) revealed that gene expression profiles of *max1-1* showed a negative correlation with those of previously reported low phosphate condition [26]. Collectively, our results show that SLs function as key regulators in the perception of phosphate deficiency and subsequent signaling.

## Materials and Methods

### Plant Materials and Growth Conditions

All WT and mutant lines used in this report were derived from *Arabidopsis thaliana* ecotype Columbia Col-0. *max1-1* and *max2-1* mutants are in the Col-0 background. *Arabidopsis* seeds were surface-sterilized and grown on *Arabidopsis* culture agar media (5 mM KNO_3_, 1 mM MgSO_4_, 1.5 mM Ca(NO_3_)_2_, 1 mM NH_4_Cl, 50 μM Fe-EDTA, 46 μM HBO_3_, 10 μM MnSO_4_, 0.77 μM ZnSO_4_, 0.32 μM CuSO_4_, 0.58 μM Na_2_MoO_4_, 0.25 μM NH_4_VO_3_, 0.7% agar) supplemented with or without 1 mM KH_2_PO_4_. The agar concentration used here contains approximately 4.0 ± 0.2 μM phosphorus in the final medium, determined by the phosphomolybdate method. Plants were cultivated at 23°C under continuous light after an initial chilling period (4°C for two days). For SL application experiments, we used the GR24, which is the most popular and widely used synthetic SL analog. All experiments were performed with the exception of plants showing abnormal growth such as extreme growth delay.

### RNA Preparation and Gene Expression Analysis

Total RNA was isolated and purified from the roots of three-week-old plants using Plant RNA Isolation reagent (Invitrogen, USA). For RNAseq analysis, the quality of total RNA was evaluated using the Agilent 2100 Bioanalyzer (Agilent, USA). Two micrograms of total RNA from roots of three-week-old plants of the WT and *max1-1* were used to make separate libraries using TruSeq RNA and TruSeq DNA Sample Prep kits as according to the manufacturer’s instructions (Illumina, USA). The quality of each library was assessed using the Agilent 2100 Bioanalyzer (Agilent) and then sequenced using an Illumina HiSeq 2000 Sequencer (paired-end sequencing, 100 bp). The data sets supporting the results of this article are available in the DDBJ database (accession: DRA001683). Data analysis was performed using the CLC Genomics Workbench (version 6.5). The experiment was performed twice (WT) or three times (*max1-1*) using independently prepared *Arabidopsis* seedlings. The complete list of regulated genes is provided in S1 Table. Common genes significantly up- or down-regulated between this experiment and previously reported experiments on phosphate deprivation [26] were plotted according to the log_2_ signal ratio (SR). The Spearman’s rank-order correlation coefficient (SCC) of SR was used to estimate the relationship between these experiments.

For the comparison of gene expression profiles between this RNAseq analysis and DNA microarray experiments, we used the DNA microarray analysis tool, AtCAST (http://atpbsmd.yokohama-cu.ac.jp/cgi/network/home.cgi) [27]. RNAseq data (WT vs. max1-1) was used as query data. Experiments showing correlations (|Spearman’s correlation coefficient| > 0.3) with query were detected.

For qRT-PCR analysis, cDNA synthesis was performed using the PrimeScript RT reagent Kit and gDNA eraser (Takara, Japan). qRT-PCR was performed on a Takara Thermal Cycler Dice Real Time System using a SYBR premix and Ex Taq (Takara). Specific primers used for qRT-PCR are listed in S2 Table. The expression of UBC (encoding ubiquitin C) was used as an internal standard.

### Determination of Root Hair Length

Plants were grown on *Arabidopsis* culture agar media for 5 days. Each seedling was transferred to fresh agar containing 1 μM GR24 or 1 mM KH_2_PO_4_. After 5 days, root hair length was measured using a stereomicroscope (SZX12, Olympus, Japan). Measurements were performed using at least ten images per treatment, employing the software IMAGEJ (http://imagej.nih.gov/ij/download.html); 20–30 root hairs were measured per image.

### Detection and Quantification of Acid Phosphatase Activity

Plants were grown on *Arabidopsis* culture agar media with or without GR24 for 10 days and then stained for acid phosphatase activity according to Lei et al. (2011) [28]. Seedlings were transferred to 0.5% (w/v) agar containg 0.01% (w/v) 5-bromo-4-chloro-3-indolyl phosphate p-toluidine salt (BCIP). After blue color development, photographs were taken using a camera attached to a stereo-microscope (SZX12, Olympus, Japan). Acid phosphatase activity was quantified as described by Richardson et al. (2001) [29]. Seedlings (4 to 6 replicates, six seedlings per replicate) were transferred to 1.0 ml of 15mM MES buffer (pH5.5) containing 0.5mM CaCl_2_ and 10 mM *p*-nitrophenyl phosphate (*p*NPP) and incubated at 25°C for 2 h. reactions were terminated by the addition of an equal volume of 0.25 M NaOH and activity was calculated from the production of *p*-nitrophenol, as determined spectrophotometrically at 405 nm relative to standard solutions.

### Determination of Metal Concentration and Anthocyanin Content

The basal stems of three- or four-week-old plants were collected and anthocyanin content was measured as described previously [30]. Anthocyanin was extracted by incubating at least ten seedlings (three or more replicates) in 300 μl of extraction solution (methanol containing 1% HCl) overnight at 4°C. After the extraction, 200 μl of water and 200μl of chloroform were added, and the mixture was centrifuged. The amount of anthocyanin was calculated as A_530_. For measurement of Pi content, seedlings grown on *Arabidopsis* culture agar media containing 100 μM phosphate were collected and dried at 80°C for 48 h. Samples were weighed and digested with 3 ml of 13 M HNO_3_ at 220°C for 1 h using MARS Xpress (CEM, USA). The plants were digested in six replicate subsamples from each replication. After digestion, the samples were collected, diluted to 5 ml, and analyzed using inductively coupled plasma–atomic emission spectroscopy (ICP–OES, Seiko, Japan). The total metal concentration of each plant was then calculated.

## Results

### Effect of SL on Phosphate Starvation Responses

**Fig 1:**
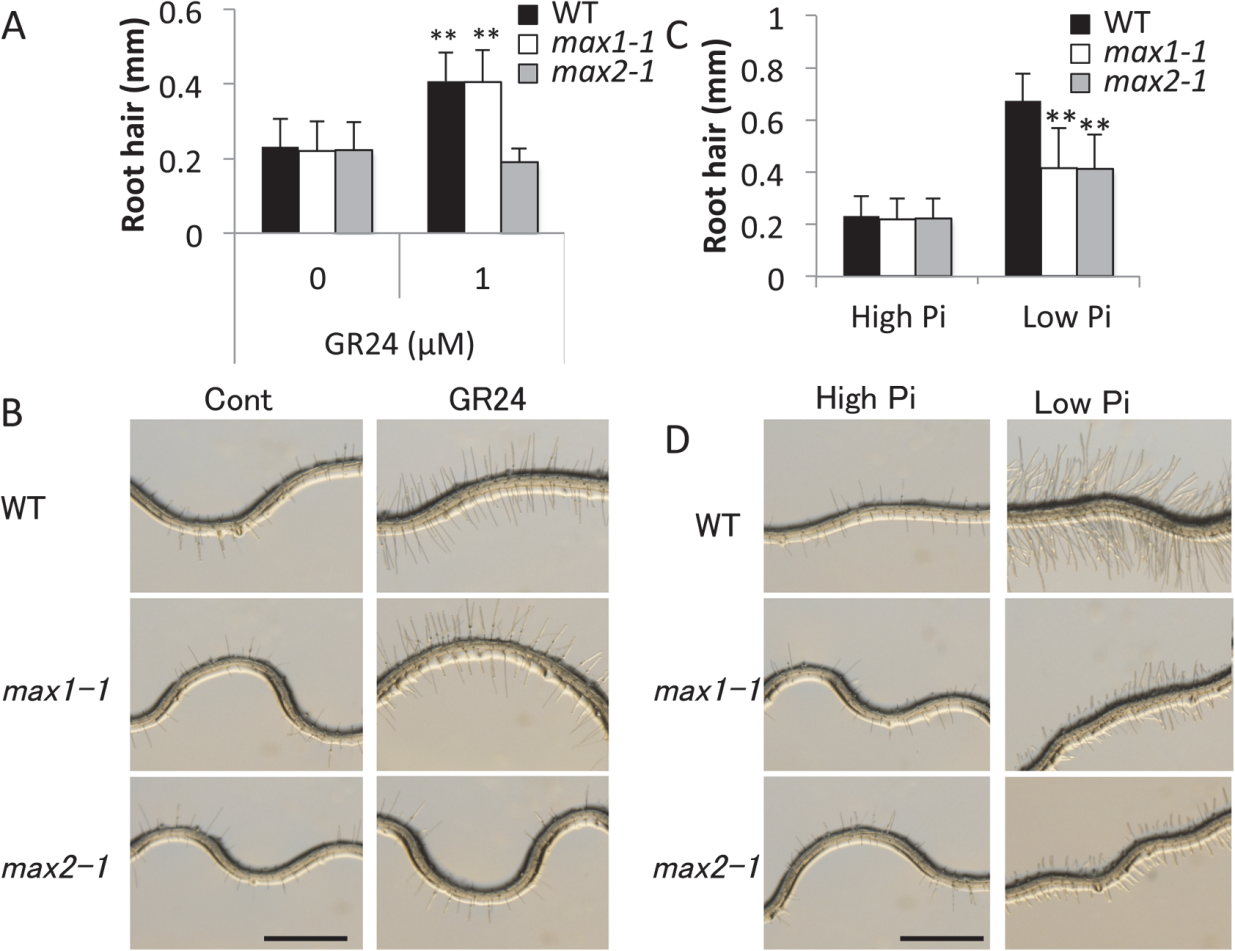
Comparison of Root Hair Length in 10-Day-Old Seedlings of WT, *max1-1*, and *max2-1* Plants. **(A, B)** Root hair length of control (0 μM) and GR24-treated (1 μM) plants. **(C, D)** Root hair length of WT and SL-mutants grown under low- and high-Pi conditions. Scale bar = 1 mm. Data presented in panels A and C are the means ± SD of 10 biological replicates. Two independent experiments were performed with similar results. Columns marked with ** indicate significant differences (Student’s t-test, *P* < 0.01). WT Columbia-0 (Col-0), *max1-1*, and *max2-1* plants are shown as black, white, and grey-filled bars, respectively.

During the early stages of root development, SLs were shown to be involved in root hair development under low-Pi conditions [25]. To better understand the effect of low Pi (10 μM) on root hair development, we measured the length of root hairs in mutants defective in SL biosynthesis and signaling (e.g., *max1-1* and *max2-1*, respectively). The synthetic SL analog, GR24, was used in these studies. In contrast to the WT Arabidopsis Col-0 and *max1-1* seedlings, root hair length was reduced in *max2-1* plants treated with 1 μM GR24 (*P* < 0.01) under high-Pi (1 mM) condition (Fig. 1A and B). The length of root hairs was similar for WT, *max1-1*, and *max2-1* seedlings grown on high-Pi (1 mM) plates; however, root hair length was significantly reduced (*P* < 0.01) in mutant seedlings cultivated under low-Pi (10 μM) conditions (Fig. 1C and D). The results obtained with the mutants indicate that SLs are key regulators of root hair development in the vegetative stage.

**Fig 2:**
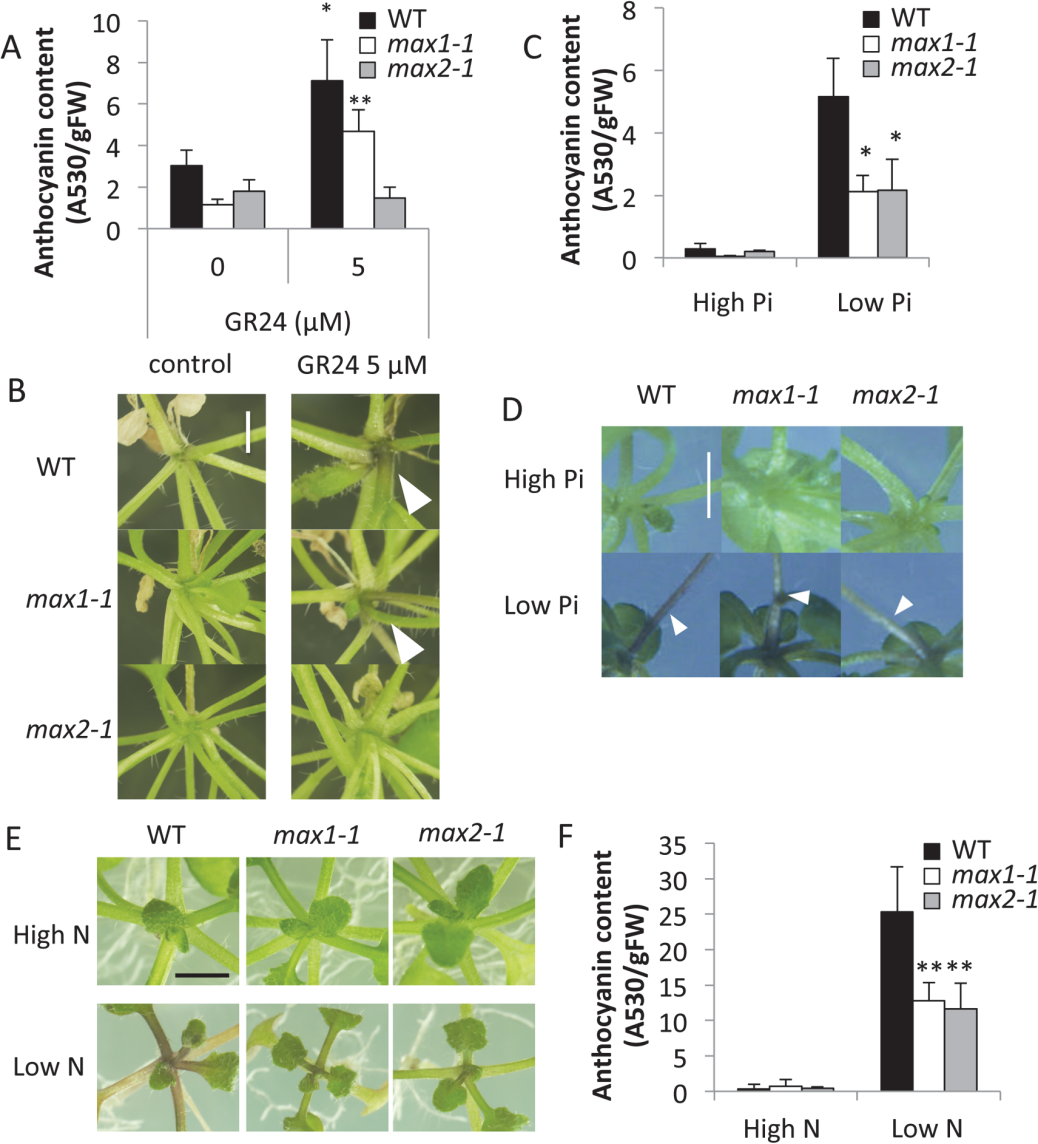
Comparison of Anthocyanin Accumulation in WT, *max1-1*, and *max2-1 Arabidopsis* Plants. **(A, B)** Anthocyanin accumulation in four-week-old plants in the absence (0 μM) or presence (5 μM) of GR24. **(C-F)** Anthocyanin accumulation in three-week-old SL-deficient plants grown under low- and high-Pi (C, D), and low-N (F). Scale bar = 0.2 cm. Data presented are the means ± SD of 4 biological replicates (each replicate contained ten or more plants). Two independent experiments were performed with similar results. * and ** indicate significant differences with respect to plants grown under conditions of high Pi (t-test, *P* < 0.05 and *P* < 0.01, respectively). Black, white, and grey bars depict WT, *max1-1*, and *max2-1* plants, respectively.

Pi starvation is known to induce anthocyanin production; thus, we examined the effect of GR24 on anthocyanin accumulation. Under high-Pi condition, application of GR24 (5 μM) significantly increased anthocyanin levels near the basal stem in WT and *max1-1* plants, but not in *max2-1*, which indicates the importance of SL signaling in anthocyanin accumulation (Fig. 2A and B). We also examined the effects of SL signaling on anthocyanin accumulation in plants grown under low-Pi (10 μM) conditions. When grown on low-Pi medium for 3 weeks, the basal stems of the WT turned dark purple, whereas the basal stems of *max1-1* and *max2-1* were pale purple (Fig. 2D). Quantitative analysis showed that the anthocyanin content in *max1-1* and *max2-1* seedlings was about 50% lower than in the WT under low-Pi conditions (Fig. 2C). Given that anthocyanin accumulation is caused by various biotic and abiotic stresses, we checked the effect of low nitrogen on anthocyanin levels. Similar to the results obtained with low Pi, the basal stems of WT seedlings showed purple coloration and substantial anthocyanin production when N was limiting, whereas anthocyanin accumulation in *max1-1* and *max2-1* was significantly reduced (*P* < 0.01) relative to the WT (Fig. 2E, F). Although it has been reported that anthocyanin accumulation is controlled by signaling pathways specific to different stressors [31], these results suggest that SLs modulate anthocyanin accumulation under conditions where both Pi and nitrogen are limited.

**Fig 3:**
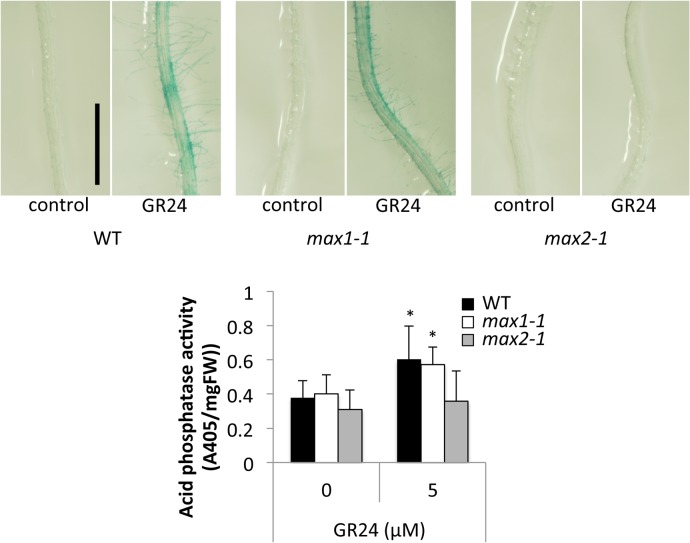
Effects of GR24 on Acid Phosphatase Secretion in 10-Day-Old Seedlings. **(A)** Acid phosphatase activity on root surfaces as detected by treatment with 5-bromo-4-chloro-3-indolyl phosphate *p*-toluidine salt (BCIP). Scale bar = 5 mm. **(B)** Acid phosphatase activities as detected by treatment with *p*-nitrophenylphosphate (*p*NPP). Data are the means ± SD of 6 (WT and *max2-1*) and 5 (*max1-1*) replicates (each replicate contained six or more seedlings). Three independent experiments were performed with similar results. * indicates significant differences from control plants (Student’s t-test, *P* < 0.01).

Under low-Pi conditions, plants increase the synthesis and secretion of acid phosphatase to acquire internal or external organic phosphate. To determine whether SL signaling plays a role in inducing acid phosphatase, we grew WT, *max1-1*, and *max2-1* seedlings on agar medium containing GR24 under high-Pi condition. Acid phosphatase activity was determined by transferring the plants onto agar plates containing 5-bromo-4-chloro-3-indolyl phosphate (BCIP), which is a substrate of acid phosphatase. GR24-treated roots of WT and *max1-1* seedlings turned blue, which indicates the induction of acid phosphatase secretion; however, *max2-1* roots did not change color when GR24 was added to the medium (Fig. 3A). The quantification of acid phosphatase activity in control and GR24-treated seedlings supported the qualitative observations and revealed the induction of SL-dependent acid phosphatase secretion (Fig. 3B).

**Fig 4:**
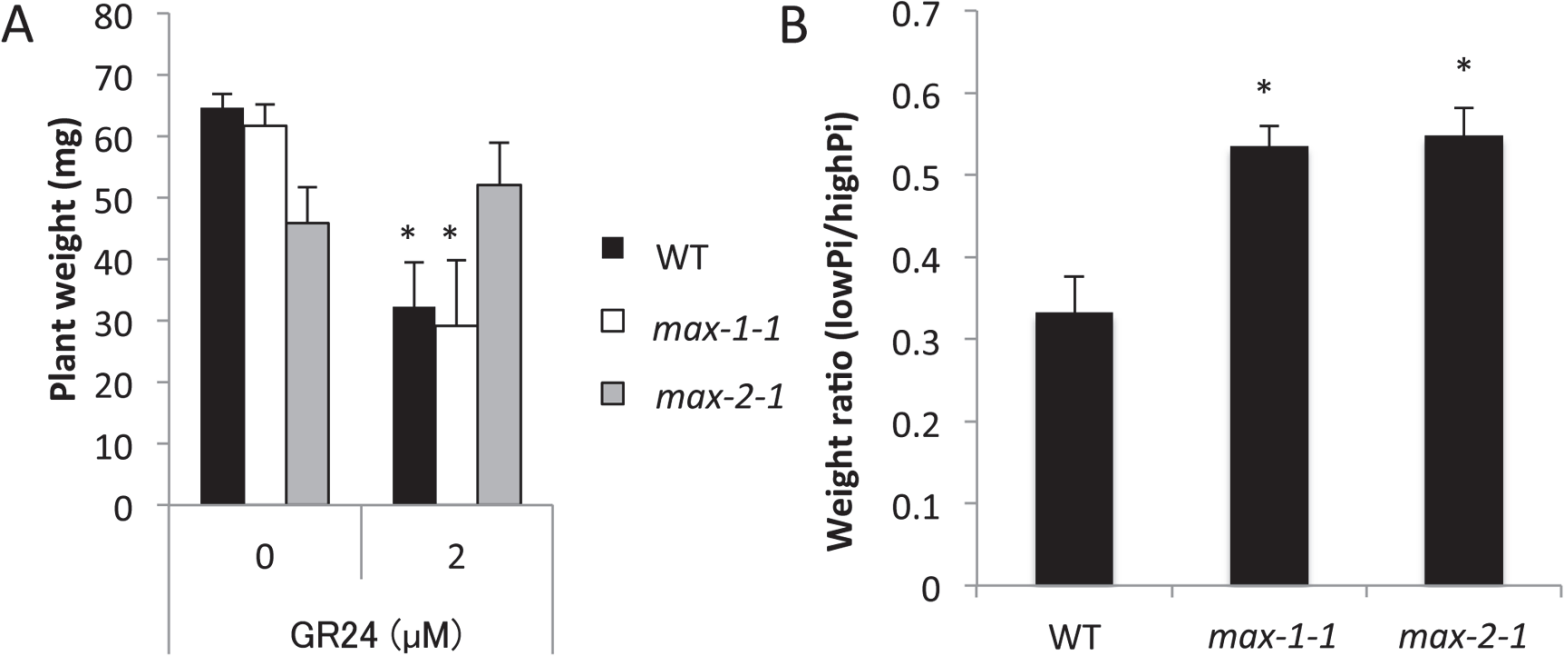
Comparison of Whole Plant Weight in WT, *max1-1*, and *max2-1* Lines. **(A)** Effect of GR24 on whole plant weight under high-Pi condition. **(B)** Ratio of plant weight of *max1-1* and *max2-1* grown under low- and high-Pi conditions. Data are the means ± SD of five biological replicates (each replicate contained six or more seedlings). Three independent experiments were performed with similar results. * indicates significant differences from control plants (A) and WT (B) (Student’s t-test, *P* < 0.01). Black, white, and grey bars represent WT, *max1-1*, and *max2-1* plants, respectively.

A reduction in whole plant weight is characteristic of the Pi starvation response [31]. To determine whether SL signaling modulates plant weight, we measured the whole plant weight of WT, *max1-1*, and *max2-1* seedlings under high-Pi (1 mM) and low-Pi (100 μM) conditions. SL treatment reduced the plant weight in WT and *max1-1* seedlings even under high-Pi conditions, whereas the plant weight of *max2-1* seedlings remained unaffected by SL application (Fig. 4A). It is important to note that the ratio of whole plant weight under low- and high-Pi conditions was significantly increased in *max1-1* and *max2-1* seedlings as compared with the WT (Fig. 4B). Thus, the growth of the SL biosynthesis and signaling mutants was less impacted by the Pi level than in WT seedlings. The results suggest that both SL biosynthesis and signaling are required for the growth defect under conditions of low Pi.

### Expression Analysis of Phosphate Starvation and SL-Induced Genes

**Fig 5:**
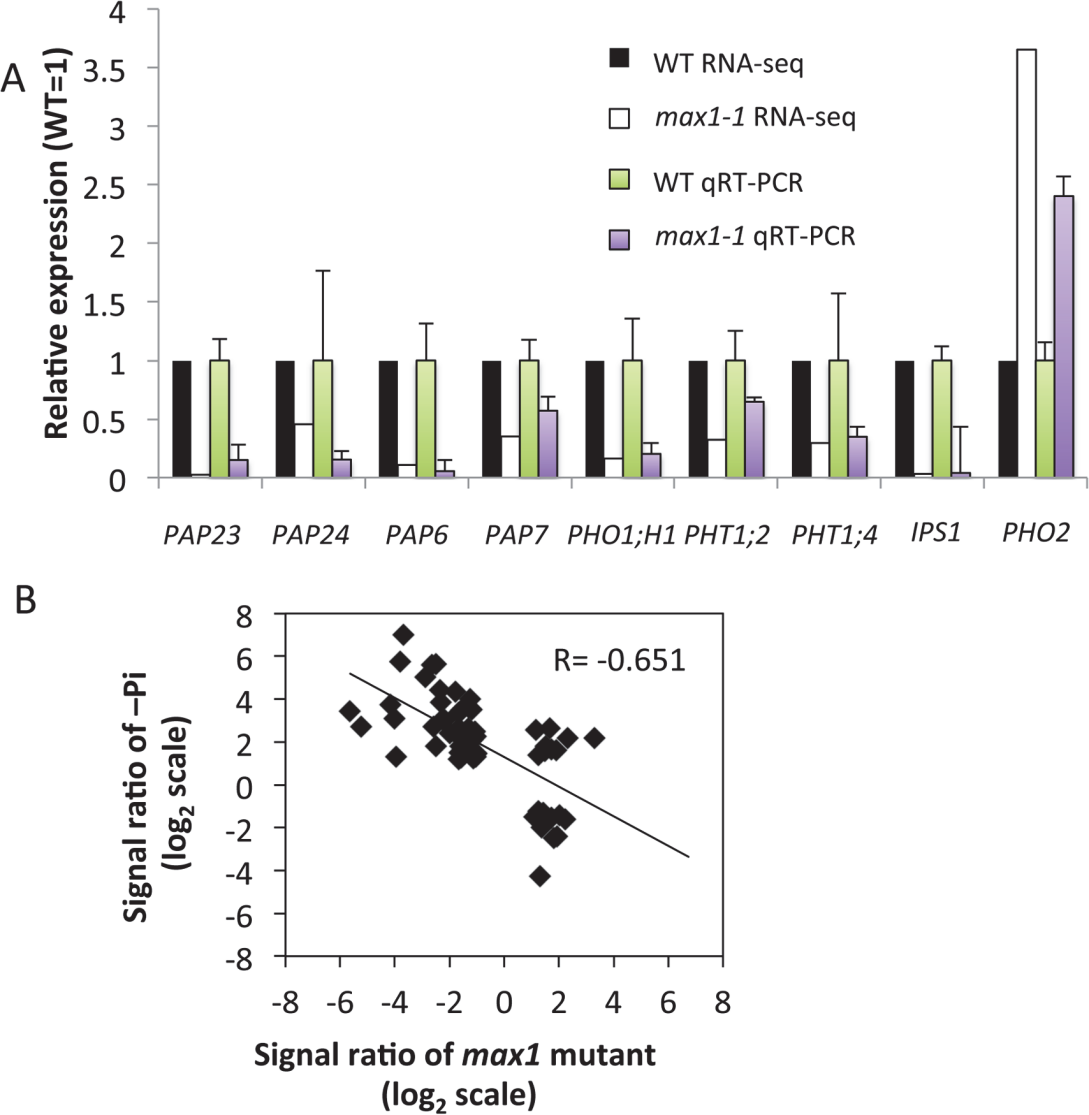
Gene Expression Analysis in WT and *max1-1* Roots. **(A)** Expression levels of PSI genes altered in *max1-1* roots. The expression level of each transcript is displayed relative to the expression level in the WT, which was assigned a value of 1. Black and white bars indicate the results of RNA-seq analysis. Green and purple bars indicate the results of qRT-PCR. The UBC gene was used as a reference in qRT-PCR. **(B)** Scatterplots showing the results of two experiments, “*max1* mutant (*max1-1* mutants compared with WT)” and “-Pi (roots of 10-day-old plants grown on a Pi-deprived plate compared with mock treatment)”. The x and y axes indicate transcript responses as a log signal ratio (mutant or treatment/WT or mock, respectively). Gene expression response of *max1-1* and Pi responsive genes were plotted. Fisher’s exact test indicates the significant correlation between “*max1* mutant” and “-Pi” (P < 0.001).

The above results suggest that SL signaling plays a role in the response of *Arabidopsis* to phosphate starvation. To examine this possibility, we performed global expression analysis using RNAseq. Three-week-old roots of WT and *max1-1* seedlings grown under high-Pi condition were collected, and RNAseq was performed. A total of 707 342 980 reads were generated using an Illumina HiSeq 2000 sequencer. Each sample was represented by an average 141 million reads (S3 Table). For each sample, 92% of the reads could be mapped to the *Arabidopsis* genome reference, and 98% of the mapped reads matched uniquely. Statistical and two-fold cut-offs were used to identify genes that were significantly and differentially expressed between WT and *max1-1*. We identified 502 down-regulated and 805 up-regulated genes in *max1-1* (Table 1, S1 Table). In the subset of identified genes, the expression of phosphate transporters (*PHO1;H1*, *PHT1;2*, and *PHT1;4*), *PAPs* and *IPS1* was down-regulated in *max1-1* (Fig. 5A), whereas *PHO2* was up-regulated in this mutant (Fig. 5A). The reliability of our RNA-seq data was validated by examining the expression of several PSI genes by qRT-PCR (Fig. 5A). To compare the expression patterns obtained in our study and a previous study focused on phosphate deprivation, we examined the Affimetrix ATH1 microarray data reported by Misson et al. (2005) [26]. Misson previously reported 302 PSI genes, and 65 of these genes (21.5%) were regulated in our study. In the set of genes previously shown to be dramatically altered during phosphate starvation (>10-fold, 35 genes; <1/10-fold, four genes) [26], we identified 15 repressed genes (43%) and one induced gene (25%) in our experiments. Thus, our results indicate a negative correlation between *max1*-regulated and PSI genes (*r* = -0.651) (Fig. 5B).

**Table 1:** Genes induced (UP) and repressed (DOWN) in *max1-1* mutant (TOP20).

Up–regulated genes AGI code	description	GO Biological process	Fold change	p value	Down–regulated genes AGI code	description	GO Biological process	Fold change	p value
AT3G55790	unknown protein	cellular response to hypoxia	25.1473383	0.024606129	AT2G23985	unknown protein	unknown	-1151.646606	0.000111291
AT5G55020	Member of the R2R3factor gene family (MYB120)	pollen tube growth	16.55878377	0.004646885	AT2G34210	Transcription elongation factor (SPT5-1)	DNA-dependent transcription	-81.91432039	0.000281985
AT4G10860	unknown protein	unknown	14.56467595	0.006941624	AT5G25980	Myrosinase (TGG2)	defense response	-51.89471001	0.022692782
AT4G10510	Subtilase family protein	metabolic process	12.89418822	0.010392075	AT5G09570	Cox19-like CHCH family protein	response to phosphate starvation	-48.11647869	0.029515311
AT1G21550	Calcium-binding EF-hand family protein	unknown	12.81848103	0.00128757	AT5G26000	member of Glycoside Hydrolase Family 1 (TGG1)	defence response	-37.72780429	0.000472743
AT4G09110	RING/U-box superfamily protein	response to zinc ion	12.51106797	0.040560229	AT3G54530	unknown protein	unknown	-36.16015257	0.046030879
AT5G56080	NICOTIANAMINE SYNTHASE2 (NAS2)	cellular response to iron ion	12.37657986	0.037883023	AT4G33710	CAP superfamily protein	unknown	-34.49263435	0.03414496
AT2G07000	unknown protein	unknown	12.11065807	0.01818548	AT2G22980	serine carboxypeptidase-like 13 (SCPL13)	proteolysis	-32.74728257	0.03371921
AT2G46494	RING/U-box superfamily protein	unknown	10.97726348	0.024396468	AT3G09922	INDUCED BY PHOSPHATE STARVATION1 (IPS1)	response to phosphate starvation	-32.50618228	0.010361506
AT2G31420	DNA binding	regulation of transcription	10.60417067	0.002511427	AT2G30770	CYP71A13	camelexin biosynthetic process	-30.53445507	0.038447844
AT1G26410	FAD-binding Berberine family protein	cellular response to hypoxia	10.40559523	0.034229354	AT2G44070	NagB/RpiA/CoA transferase-like superfamily protein	cellular metabolic process	-27.28442026	0.039138937
AT5G52300	LOW-TEMPERATURE-INDUCED 65 (LTI65)	leaf senescence	10.33166331	0.043559123	AT2G47520	HYPOXIA RESPONSIVE ERF (HRE2)	response to anoxia	-24.73233108	0.006948727
AT5G50140	Ankyrin repeat family protein	unknown	9.943750279	0.026171042	AT3G25240	unknown	protein unknown	-24.15183632	0.003700508
AT2G23190	CYP81D7	oxidation-reduction process	9.427410954	0.019394508	AT3G55240	overexpression leads to PEL phenotype	developmental process	-22.18062111	0.000494586
AT5G05280	DEFECTIVE IN ANTHER DEHISCENCE1-ACTIVATING FACTOR (DAF)	anther dehiscence	9.326825512	0.042281532	AT2G39030	N-ACETYLTRANSFERASE ACTICITY 1 (NATA1)	response to jasmonic acid stimulus	-20.93344909	0.032728491
AT4G14368	Regulator of chromosome condensation family protein	unknown	9.11537737	0.003409135	AT2G25450	encodes a protein whose sequence is similar to ACC oxidase	glucosinolate biosynthetic process	-20.84708869	0.021694602
AT2G19500	CYTOKININ OXIDASE2 (CKX2)	cytokinin catabolic process	8.794091084	0.036640016	AT2G23270	unknown protein	cellular response to hypoxia	-20.46464874	0.006153645
AT3G47480	Calcium-binding EF-hand family protein	defense response	8.779090678	0.011126662	AT4G13700	purple acid phosphatase 23 (PAP23)	acid phosphatase activity	-18.57170024	0.000166861
AT2G15042	Leucine-rich repeat family protein	unknown	8.351497669	0.032278236	AT1G08165	unknown protein	unknown	-18.49418393	0.035072942
AT3G52970	CYP76G1	oxidation-reduction process	8.241920159	0.043883855	AT1G10640	Pectin lyase-like superfamily protein	carbohydrate metabolic process	-17.95259434	0.00375611

SLs function in various developmental and environmental processes including shoot branching, leaf senescence, and pathogen infection [12,32]. Furthermore, crosstalk has been shown to exist between SLs and other plant hormones, including IAA, cytokinin, ethylene, gibberellin, brassinosteroids, abscisic acid, and methyl jasmonate [32–38]. To compare the relationship between SLs and other signaling compounds that modulate gene expression, we analyzed our RNAseq data using AtCAST, a tool designed for Arabidopsis DNA microarray data [27]. AtCAST can be used to identify co-regulated networks between DNA microarray experiments based on module-based correlation analysis. Using AtCAST, we identified weak positive relationships between our results and senescing leaf and IAA treatments, and weak negative relationships with methyl jasmonate, t-zeatin treatment and B. cinerea infection (S1 Fig., S4 Table).

### Analysis of Metal Content

**Fig 6:**
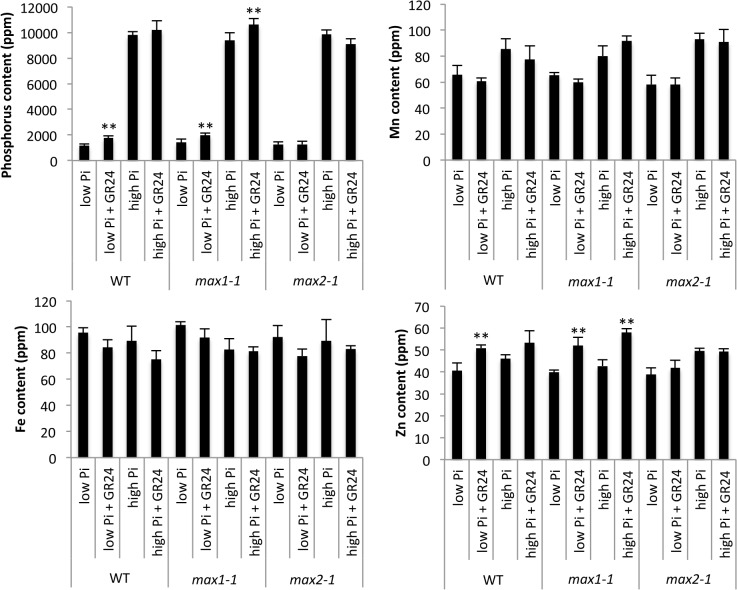
Comparison of Phosphorus and Micronutrient Content in Three-Week-Old WT and *max1-1* Seedlings Grown with High (1 mM) or Low (0.1 mM) Pi in the Presence or Absence of GR24. Data represent the means ± SD of five biological replicates (each replicate contained ten or more seedlings). ** indicates significant differences from GR24 untreated plants (Student’s t-test, *P* < 0.01).

Because the expression of some Pi transporters and PAPs were altered in *max1-1* mutants, we measured the metal content in *max1-1* and SL-treated plants grown under high-Pi (1 mM) and low-Pi (100 μM) conditions. As shown in Fig. 6, the phosphorus content of WT and *max1-1* increased in response to GR24 treatment under low Pi condition, though that of *max2-1* did not. In addition, the Zn content of WT and *max1-1* also increased in response to GR24. The concentration of other metals (Mn and Fe) remained unchanged by treatment of GR24.

## Discussion

In this study, we sought to demonstrate the relationship between SL and Pi starvation signaling. We observed morphological changes in both SL-biosynthetic (*max1-1*) and signaling (*max2-1*) mutants under high- and low-Pi conditions. SL responsive plants (WT and *max1-1*) treated by SL showed Pi-deficient phenotypes including enhanced root hair elongation, anthocyanin accumulation, acid phosphatase secretion, and reduction of plant weight even under high-Pi condition (Fig. 1A and B, Fig. 2A and B, Fig. 3 and Fig. 4A). In addition, the Pi content was significantly and slightly increased in SL responsive plants by treatment of SL under low Pi and high Pi condition, respectively (Fig. 6). Furthermore, deficiencies in SL biosynthesis and signaling repressed several responses indicative of low Pi (Fig. 1C and D, Fig. 2C and D and Fig. 4B). Our results suggest that SL signaling is involved in multiple responses to Pi starvation under both high- and low-Pi conditions, possibly by mediating and/or compensating for signaling defects caused by Pi-deficiency. Other pathways may regulate the low-Pi response because both the SL biosynthesis and signaling mutants retained weak but significant responses under the low-Pi conditions with respect to root hair elongation and the accumulation of anthocyanins (Figs 1, 2). In addition, we used the different concentrations of GR24 and Pi, as the sensitivities of GR24 and Pi in each assay were different in our preliminary tests. Possibly, effects of GR24 and Pi may vary with their concentration.

It is known that GR24 could increase abiotic stress resistance in Arabidopsis [37]. As shown in Fig. 4B, the weight ratio (low Pi/high Pi) was increased in SL biosynthesis (*max1-1*) and signaling (*max2-1*) mutants. In addition, GR24 reduced the weight in WT and *max1-1* under high Pi condition (Fig. 4A). As reduction of plant weight was considered as a strategy to adapt to the low-Pi condition, these results suggest that SLs could also increase low-Pi resistance in Arabidopsis.

In contrast to the reduced expression of *PHT*s (*PHT1;2*, *PHT1;4*) and *PAP*s (*PAP6*, *PAP7*, *PAP23*, *PAP24*), the expression of *PHO2* in *max1-1* was increased. *PHO2* encodes ubiquitin-conjugating enzyme UBC24, which regulates protein degradation and gene expression of *PHT*s and *PHO1* [4,39]. As the expression of *PHO2* is necessary to maintain Pi homeostasis, SLs may modulate the signals induced by low-Pi conditions to regulate *PHO2* expression. In addition, the expression level of CHS and FLS was reduced in *max1-1* in accordance with the results of anthocyanin accumulation, though that of CHI was not (S5 Table). Expression analysis was performed using genes in the root, while anthocyanin content was estimated using pigments in the shoot. This might be the reason why the expression of CHI was up-regulated in *max1-1*.

The defect in SL signaling might impact other responses in addition to those associated with Pi starvation. For example, regarding anthocyanin accumulation, nitrogen starvation mimicked P starvation (Fig. 2). It has been reported that nitrogen deficiency promotes the production of SLs in sorghum [40]. In *Arabidopsis*, there may be a regulatory network for anthocyanin accumulation controlled by SLs under nitrogen-deficient conditions. Furthermore, the expression pattern of *max1-1* showed a weak correlation with several stress responses (S1 Fig.); thus, SLs may be involved in modulating various stress signaling pathways that are associated with anthocyanin accumulation.

SLs modulate plant architecture, various defense responses, and salt stress through interactions with other plant hormones including auxin, cytokinin, ethylene, jasmonic acid, and abscisic acid [12,32–34,37]. Our analysis of the RNAseq data with that obtained in microarray experiments of various hormone treatments (e.g., t-zeatin, IAA, methyl jasmonate) showed the existence of weak correlations between SL and hormone treatments (S1 Fig., S4 Table). Our analysis also supports previous observations in gene expression profiling [12,32–34].

**Fig 7:**
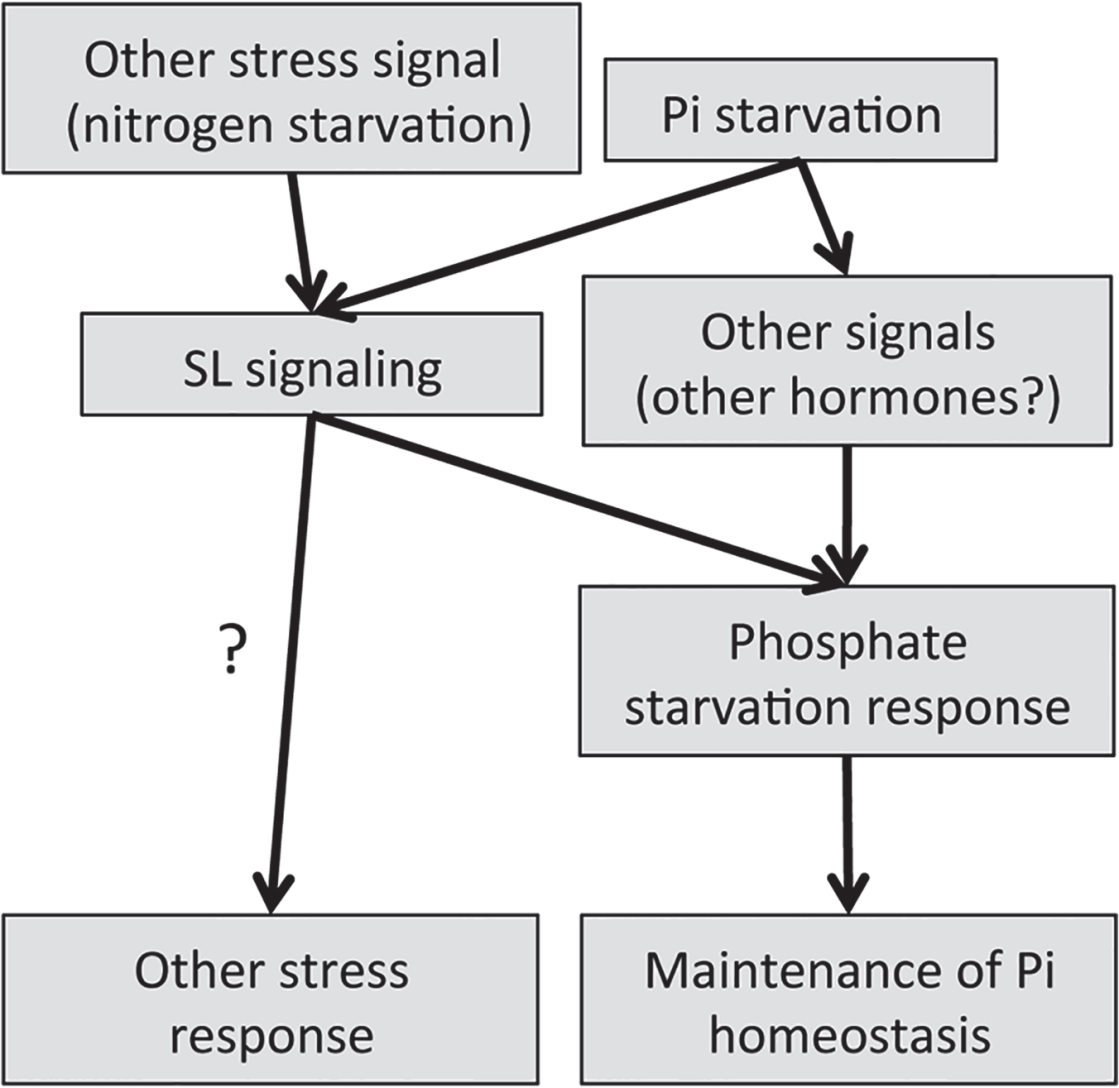
Model illustrating how SLs impact adaptation to Pi starvation. Upregulation of SL biosynthesis during Pi starvation can impact the ability to respond to low-Pi response through regulation of root hair elongation and PSI gene expression, though unknown SL independent pathways are involved in the adaptation of a low-Pi response.

Herein we propose a model to illustrate how SLs impact adaptation to Pi starvation (Fig. 7). Low-Pi conditions enhance SL production [11,24]. The increased SL levels control root hair elongation, anthocyanin accumulation, acid phosphatase secretion, plant weight, and PSI gene expression to adapt to low-Pi condition. Altered root hair elongation, acid phosphatase secretion and PSI gene expression are known to affect Pi uptake from soil and internal Pi remobilization, which enable plants to maintain Pi homeostasis when grown under low-Pi conditions. In addition, the low-Pi responses in SL-biosynthetic and signaling mutants suggest the existence of additional unidentified pathways. Given that plant hormones such as auxin, ethylene, cytokinin, abscisic acid, and gibberellin are known to influence the low-Pi response [28,30,38,41,42], it remains possible that these hormones function as key regulators of unknown pathways. Furthermore, our results show that nitrogen starvation also impacts SL signaling; thus, many questions remain unanswered regarding the role of hormones and environmental stress on SL-mediated responses. Further studies are planned to more clearly understand the complicated signaling networks that modulate the phosphate starvation response in plants.

## Supporting Information

S1 Fig Correlation analysis of gene expression experiments (RNAseq or DNA microarray) using AtCAST. Experiments showing a significant correlation (Spearman’s correlation coefficient (SCC) > 0.3) with “*max1-1* (*max1-1* mutants compared with wild-type)” were searched. Red arrows indicate strong positive relationships (SCCs > 0.7); blue arrows indicate strong negative relationships (SCCs < -0.65); pink arrows, moderate relationships (SCCs > 0.5); and light blue arrows indicate moderately negative relationships (SCCs < -0.5). Arrowhead directions indicate whether the interactions were uni- or bidirectional.
(PDF)

S1 Table Genes induced (UP) and repressed (DOWN) in max1-1 mutant. (XLSX)

S2 Table Specific primers used in this experiment. (XLSX)

S3 Table Reads and genes mapped in each generated cDNA library. (XLSX)

S4 Table List of experiments included in the module base correlation network of *max1-1* mutant. Experiments showing a significant correlation (Spearman’s correlation coefficient (SCC) > 0.3) with “max1-1 (max1-1 mutants compared with wild-type)” were searched.
(XLS)

S5 Table List and expression of anthocyanin synthesis genes. (XLSX)
