# Supplementary figures and images for: Strigolactone Regulates Anthocyanin Accumulation, Acid Phosphatases Production and Plant Growth under Low Phosphate Condition in Arabidopsis
Source: PLoS One. 2015 Mar 20;10(3):e0119724. doi: 10.1371/journal.pone.0119724 (PMC4368578; doi:10.1371/journal.pone.0119724)

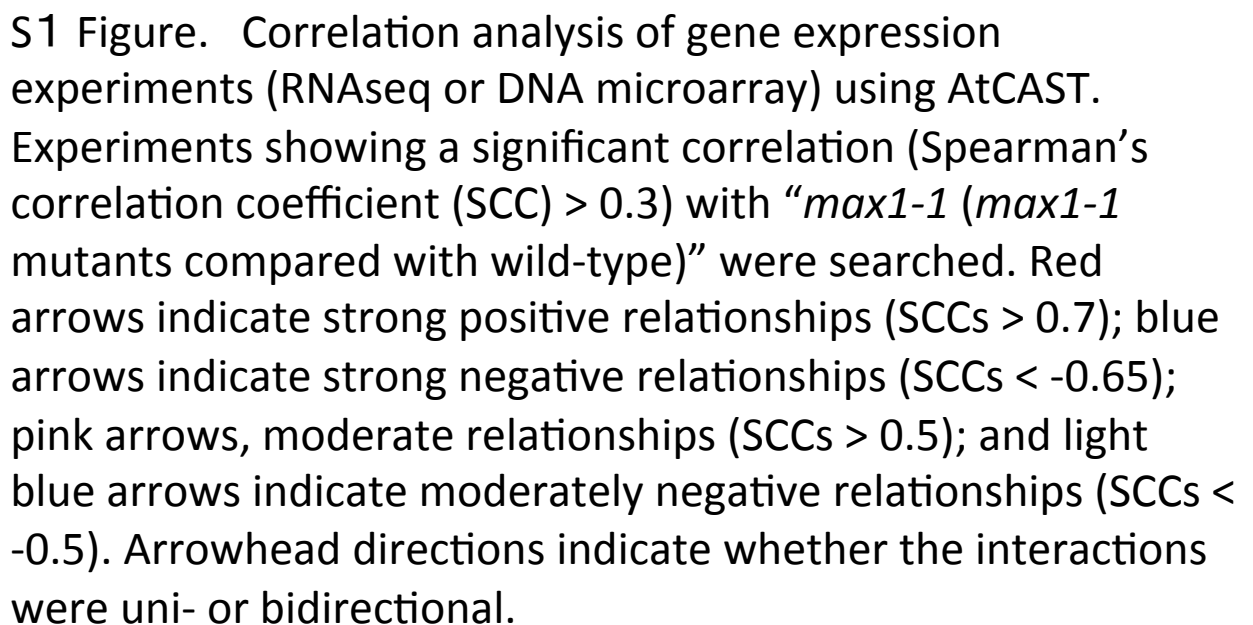

Supplement: S1 Fig — Experiments showing a significant correlation (Spearman’s correlation coefficient (SCC) > 0.3) with “max1-1 (max1-1 mutants compared with wild-type)” were searched. Red arrows indicate strong positive relationships (SCCs > 0.7); blue arrows indicate strong negative relationships (SCCs < -0.65); pink arrows, moderate relationships (SCCs > 0.5); and light blue arrows indicate moderately negative relationships (SCCs < -0.5). Arrowhead directions indicate whether the interactions were uni- or bidirectional. (PDF) [file pone.0119724.s001.pdf]
